# Cognition and transcranial sonography in Parkinson's disease patients with or without orthostatic hypotension

**DOI:** 10.1002/brb3.2252

**Published:** 2021-07-21

**Authors:** Jia‐jing Wu, Hong Jin, Ying‐qi Shao, Cheng‐jie Mao, Jing Chen, Chun‐feng Liu

**Affiliations:** ^1^ Department of Neurology The Second Affiliated Hospital of Soochow University Suzhou China; ^2^ Department of Neurology Suqian First Hospital Suqian China; ^3^ Jiangsu Key Laboratory of Neuropsychiatric Diseases and Institute of Neuroscience Soochow University Suzhou China

**Keywords:** Cognition, orthostatic hypotension, Parkinson's disease, transcranial sonography

## Abstract

**Background:**

Orthostatic hypotension (OH) is a common nonmotor symptom in patients with Parkinson's disease (PD), with an incidence ranging from 14% to 54%.

**Aims:**

This study explored changes in cognition and transcranial sonography (TCS) findings in patients with PD and OH.

**Methods:**

We enrolled PD patients who visited the outpatient or inpatient department from 2017 to 2020. Blood pressure was measured in different positions, and demographic data were collected. Motor and nonmotor symptoms were evaluated using standard scales. A subset of 107 patients underwent TCS.

**Results:**

We enrolled 66 PD‐OH patients and 92 PD‐no orthostatic hypotension (NOH) patients. There were no significant differences in gender, age, disease duration, or Hoehn and Yahr stage between groups. Binary logistic regression revealed age as an independent risk factor for OH in PD patients. There were statistically significant group differences in visuospatial and executive function and Unified Parkinson's Disease Rating Scale (UPDRS) I and II scores (*p* < .05). Among PD‐OH patients, there was a statistically significant difference in UPDRS II and III scores between patients with or without clinical symptoms (*p* < .05). The substantia nigra (SN) area was significantly larger in PD‐NOH patients (0.45 ± 0.18 cm^2^) than PD‐OH patients (0.34 ± 0.16 cm^2^) (*p* < .05).

**Conclusions:**

PD‐OH patients had poorer visuospatial and executive function and lower UPDRS I and II scores compared with PD‐NOH patients. Within the PD‐OH group, there was no significant difference in cognition between patients with or without clinical symptoms. The difference in the SN area may indicate different subtypes of PD or a tendency to develop parkinsonism syndrome.

## Abstract 2

PD patients with OH have poorer visuospatial and executive function and life quality. What's more, symptomatic OH have higher scores in motor sympotoms and daily life. Finally, PDOH patients have smaller substantia nigra area in transcranial sonography.

## 1 INTRODUCTION

Orthostatic hypotension (OH) is a nonmotor symptom in Parkinson's disease (PD) that is more common in advanced stages of the disease. OH reflects autonomic nervous system dysfunction, and the prevalence in PD is about 53% (Zhang et al., 2019). This condition is associated with many other nonmotor symptoms such as lightheadedness and fatigue (Xin et al., 2016). Lewy bodies are a pathologic biomarker of PD, and alpha‐synuclein is an important component of these proteinaceous aggregates. Alpha‐synuclein is deposited in both the central nervous system and peripheral autonomic nervous system. Central and peripheral baroreflex mechanisms are damaged in PD‐OH patients (Freeman et al., 2018; Goldstein et al., 2005). Levodopa, dopamine receptor agonists, and other PD treatments may cause or aggravate OH (Kujawa et al., 2000). This nonmotor symptom is burdensome to PD patients and their family because it can cause repeated falls, and current interventions are inadequate. According to a population‐based, prospective, longitudinal cohort study, only 0.5% of PD patients use antihypertensive drugs (Hiorth et al., 2019). There is an urgent need for research focused on the incidence, clinical features, and interventions for OH in PD patients.

Cognitive dysfunction is another common nonmotor symptom in PD. A systematic review reported that the point prevalence of dementia in PD was 24%–31% (Aarsland et al., 2005). Executive, attentional, and visuospatial dysfunction and memory impairment are common in patients with PD dementia (Aarsland et al., 2017). Long disease duration and atypical neurological features such as early autonomic failure are risk factors for cognitive dysfunction in PD (Hanagasi et al., 2017). OH is also associated with cognitive impairment (McDonald et al., 2016). A previous study reported that patients with PD‐OH exhibit transient deficits in executive function, memory, and visuospatial function in an upright position, but performance improves in the supine position (Zhang et al., 2019). Dementia in PD decreases the quality of life of both patients and their caregivers and increases healthcare‐related costs (Svenningsson et al., 2012). Investigating OH may help clarify the mechanisms underlying PD dementia and identify novel targets to improve cognition in PD patients.

Transcranial sonography (TCS) is a noninvasive method for PD diagnosis. Patients with PD show significant lager substantia nigra (SN) area in TCS compared to normal controls (Tao et al., 2019). An echogenic area between 0.20 and 0.25 cm^2^ occurs in the SN of 90% of PD patients (Berg et al., 2013). This may be associated with abnormal deposition of iron‐containing protein. OH is directly associated with atrophy of subcortical structures, particularly the caudate. The striatum is also affected by blood pressure (BP) variabilities (Yoo et al., 2020). Study show that in healthy individuals, hyper echogenicity SN has been associated with slight deficits in specific cognitive functions (Yilmaz et al., 2016). We would like to explore the difference of TCS image between patients with and without OH.

In our study, we explored the clinical characters, motor symptoms, and nonsymptoms in PD patients with or without OH. Furthermore, we evaluated TCS image characteristics to find the probable mechanisms of OH.

## 2 METHODS

### 2.1 Participants

A total of 158 PD patients treated at the Second Affiliated Hospital of Soochow University from 2017 to 2020 were included in the study. All patients were diagnosed with PD according to the diagnostic criteria of the UK Parkinson's Disease Society Brain Bank by at least two neurologists (Hughes et al., 1992). Of these, 107 patients underwent TCS. We excluded PD patients who were taking the other chronic medications. This study was approved by the ethics committee of the Second Affiliated Hospital of Soochow University.

### 2.2 Study design

This was a cross‐sectional study. We used a corrected electronic sphygmomanometer to measure BP. Measurements were taken after 5 min in a supine position and 3 min in an orthostatic position. OH was defined as a decrease of at least 20 mm Hg in systolic BP (SBP) and/or 10 mm Hg in diastolic BP (DBP) within 3 min from shifting from the supine to upright position. Upright mean blood pressure (MBP) < 75 mm Hg is highly specific and sensitive for identifying symptomatic OH (Palma et al., 2015). According to OH and MBP, we divided patients into three groups: including PD patients without OH (PD‐[neurogenic orthostatic hypotension] NOH), asymptomatic PD‐OH, and symptomatic PD‐OH. Demographic data were collected for all patients.

PD‐related symptoms were evaluated by trained clinicians. Cognition was evaluated with the Mini‐Mental State Examination (MMSE) and Montreal Cognitive Assessment (MoCA) including visuospatial/executive functions, naming, attention, learning, abstraction, delayed verbal memory, and orientation. Motor symptoms were assessed with the Unified Parkinson's Disease Rating Scale (UPDRS III). Nonmotor symptoms were evaluated by nonmotor symptom questionnaire (NMSQ). Emotional problems were measured with the Hamilton Depression Scale (HAMD)−24 items and the Hamilton Anxiety Scale (HAMA)−14 items. Daily life was assessed with the UPDRS II and Parkinson's Disease Quality of Life Questionnaire (PDQL)−39. Fatigue was measured with the Fatigue severity scale (FSS).

TCS was performed as previously described (Sheng et al., 2017). It is according to the standardized procedure of TCS in neurodegenerative diseases established on the Ninth Meeting of the European Society of Neurosonology and Cerebral Hemodynamics (Berg et al., 2008; Walter, Behnke, et al., 2007 ). A 2.5‐MHz sonographic device (Sequoia 512, Siemens Medical Solutions USA, Inc. 4V1C transducer) was used to detect both temporal bone windows. Poor temporal bone windows prevented us from detecting the image. As a result, patients with poor temporal bone windows were excluded. Reports were finished and confirmed by sonologists who received uniform training. They were blinded to the clinical diagnosis and assessment. We recorded middle cerebral artery velocity and resistance of the artery, third ventricle (V3) width, and level and area of the SN.

### 2.3 Statistical analysis

We used SPSS IBM SPSS Statistics (Version 25.0. Armonk, NY: IBM Corp) for all statistical analyses. If continuous variables were normally distributed, independent sample *t*‐tests were used; Mann–Whitney *U* tests were performed to analyze continuous variables, and these were not normally distributed. Binary logistic regression analysis was carried out to identify independent risk factors. Differences were considered significant at *p* < .05.

## 3 RESULTS

Among the 158 PD patients in the study, 66 (41.8%) had OH and 92 (58.2%) patients did not. The PD‐OH group had significantly higher supine systolic pressure (SSP), orthostatic systolic pressure (OSP), orthostatic diastolic pressure (ODP), and orthostatic mean arterial pressure (OMAP) (all *p* < .05). The PD‐OH and PD‐NOH groups did not differ in gender, age, duration, or disease stage (all *p* > .05, Table 1).

**Table 1:** Basic statistics and motor and non‐motor symptom evaluation of the Parkinson's disease‐orthostatic hypotension (PD‐OH) and PD‐no orthostatic hypotension (NOH) groups

	PD‐OH (*n* = 66)	PD‐NOH (*n* = 92)	*p* value
Male (%)	46/66 (69.7%)	59/92(64.1%)	.465
Age (years)	67.18 ± 7.95	63.20 ± 12.07	.063
Duration (years)	7.41 ± 4.21	6.27 ± 3.98	.102
H‐Y stage	2.42 ± 0.73	2.27 ± 0.96	.072
Recumbent systolic pressure (mm Hg)	140.26 ± 18.07	123.40 ± 12.33	.000*
Recumbent diastolic pressure (mm Hg)	75.18 ± 12.38	75.20 ± 10.18	.540
Orthostatic systolic pressure (mm Hg)	106.02 ± 21.53	128.37 ± 15.08	.000*
Orthostatic diastolic pressure (mm Hg)	64.79 ± 12.60	79.85 ± 9.72	.000*
Orthostatic mean arterial pressure (mm Hg)	78.45 ± 13.33	95.07 ± 10.21	.000*
MMSE	25.98 ± 4.03	26.12 ± 3.69	.838
MoCA	20.61 ± 5.33	21.91 ± 4.84	.126
Visuospatial/executive functions	2.61 ± 1.54	3.21 ± 1.47	.018*
Naming	4.92 ± 1.30	2.58 ± 0.67	.961
Attention	4.92 ± 1.30	5.04 ± 1.15	.700
Learning	1.74 ± 0.92	1.87 ± 0.99	.334
Abstraction	1.02 ± 0.64	1.16 ± 0.80	.169
5‐Minute delayed verbal memory	2.18 ± 1.80	2.29 ± 1.71	.664
Orientation	5.62 ± 0.91	5.76 ± 0.52	.811
UPDRSI	4.36 ± 2.26	3.5 ± 2.16	.240
UPDRSII	14.26 ± 6.91	12.03 ± 6.23	.047*
UPDRSIII	27.08 ± 14.74	25.37 ± 12.26	.543
NMSQ	10.59 ± 5.42	9.24 ± 6.07	.061
FSS	3.16 ± 1.80	3.16 ± 2.41	.616
HAMA	10.42 ± 7.87	9.51 ± 7.32	.455
HRSD	11.03 ± 8.44	11.62 ± 9.88	.913
PDQ‐39	37.16 ± 22.77	31.51 ± 23.49	.091

*Note*: Values are reported as the mean ± standard deviation (SD).

* *p *< .05 was considered statistically significant.

Abbreviations: FSS, Fatigue Severity Scale; H‐Y, Hoehn and Yahr; HAMA, Hamilton Anxiety Scale; HRSD, Hamilton Rating Scale for Depression; MMSE, Mini‐Mental State Examination; MoCA, Montreal Cognitive Assessment; NMSQ, Non‐Motor Symptoms Questionnaire; PDQ‐39, Parkinson's Disease Questionnaire; UPDRS, Unified Parkinson's Disease Rating Scale.

Patients with OH tended to have lower scores in visuospatial/executive functions and UPDRS II compared with the PD‐NOH group (*p* < .05). There were no significant differences in other evaluations of motor and nonmotor symptoms including MMSE, MoCA (naming, attention, learning, abstraction, 5‐minute delayed verbal memory), UPDRS I, UPDRS III, NMSQ, FSS, HAMA, Hamilton Rating Scale for Depression (HRSD), and PDQ‐39 (all *p* > .05, Table 1). We performed binary logistic regression to identify independent variables associated with the presence of OH. The results showed that age was an independent risk factor for OH (*p* = .04, odds ratio = .963; Table 2). There was no correlation between the extent of BP difference and cognitive function.

**Table 2:** Binary logistic regression results

	B	SE	Wald (Xin et al., 2016)	OR	95% CI	*p* value
Male sex	−0.36	0.363	0.985	0.698	0.342–1.421	.321
Age (years)	−0.038	0.018	4.23	0.963	0.928–0.009	.040*
Duration (years)	−0.048	0.045	1.134	0.953	0.873–1.041	.287
H‐Y stage	−0.183	0.217	0.71	0.833	0.544–1.274	.399

Abbreviations: B, unstandardized beta reported from regression analysis; CI, confidence interval; OR, odds ratio; SE, standard error.

* *p *< .05 was considered statistically significant.

In the PD‐OH group, 12 (18.18%) patients had symptomatic OH. There were no differences in age, gender, duration, or H‐Y stage between subjects with asymptomatic and symptomatic OH (*p* > .05). However, patients with symptomatic OH tended to have higher lower SDP, OSP, ODP, and MBP measurements. Symptomatic OH was also associated with higher scores for UPDRS II (*p* = .003) and UPDRS III (*p* = .016). The groups did not differ in the other assessments of motor and nonmotor symptoms (Table 3).

**Table 3:** Basic statistics and motor and non‐motor symptoms evaluation of symptomatic orthostatic hypotension (OH) and asymptomatic OH

	Symptomatic OH (*n* = 12)	Asymptomatic OH (*n* = 53)	*p* value
Male (%)	3/12 (25.0%)	17/53 (32.1%)	.894
Age (years)	68.42 ± 2.76	66.91 ± 1.04	.556
Duration (years)	7.66 ± 1.01	7.35 ± 0.59	.641
H‐Y stage	2.75 ± 0.23	2.35 ± 0.09	.098
Recumbent systolic pressure (mm Hg)	140.33 ± 3.55	140.24 ± 2.61	.987
Recumbent diastolic pressure (mm Hg)	67.33 ± 3.32	76.92 ± 1.63	.010*
Orthostatic systolic pressure (mm Hg)	75.25 ± 2.01	112.85 ± 2.34	.000*
Orthostatic diastolic pressure (mm Hg)	48.25 ± 2.70	68.46 ± 1.37	.000*
MMSE	24.41 ± 1.73	26.33 ± 0.47	.225
MoCA	18.25 ± 1.36	21.12 ± 0.73	.056
Visuospatial/executive functions	2.41 ± 0.43	2.64 ± 0.21	.615
Naming	2.50 ± 0.19	2.51 ± 0.11	.599
Attention	4.41 ± 0.36	5.03 ± 1.77	.051
Learning	1.33 ± 0.22	1.83 ± 0.12	.072
Abstraction	1.00 ± 0.21	1.02 ± 0.86	.932
Delayed verbal memory	1.50 ± 0.50	2.33 ± 0.24	.132
Orientation	5.08 ± 0.45	5.74 ± 0.09	.131
UPDRSI	5.08 ± 0.60	4.20 ± 0.31	.211
UPDRSII	19.08 ± 1.65	13.19 ± 0.91	.003*
UPDRSIII	33.91 ± 3.10	25.56 ± 2.06	.016*
NMSQ	12.33 ± 1.47	10.20 ± 0.74	.205
FSS	3.49 ± 0.59	3.08 ± 0.23	.538
HAMA	12.58 ± 2.78	9.94 ± 1.01	.449
HRSD	13.58 ± 3.63	10.46 ± 0.99	.532
PDQ‐39	40.00 ± 6.91	36.54 ± 3.08	.594

*Note*: Values are reported as the mean ± standard deviation (SD).

Abbreviations: FSS, Fatigue Severity Scale; H‐Y, Hoehn and Yahr; HAMA, Hamilton Anxiety Scale; HRSD, Hamilton Rating Scale for Depression; MMSE, Mini‐Mental State Examination; MoCA, Montreal Cognitive Assessment; NMSQ, Non‐Motor Symptoms Questionnaire; OH, orthostatic hypotension; PDQ‐39, Parkinson's Disease Questionnaire; UPDRS, Unified Parkinson's Disease Rating Scale.

* *p *< .05 was considered statistically significant.

Only 73 (33 PD‐OH, 40 PD‐NOH) patients completed TCS. Those who could not schedule the examination or had poor temporal windows were excluded. The side with larger SN area and the lower velocity middle cerebral artery were used for comparisons between groups. The level of SN was adjusted as previously described (Sheng et al., 2017). The larger area of these patients belonged to the third level. The area of SN was both ≥0.20 cm^2^, which is a cut‐off value to detect PD. We found that PD‐NOH had a larger mean SN area (*p* = .009). There were no significant differences in middle cerebral velocity or resistance or third ventricle width (Table 4).

**Table 4:** Transcranial sonography results in the Parkinson's disease‐orthostatic hypotension (PD‐OH) and PD‐neurogenic orthostatic hypotension (NOH) groups

	PD‐OH (*n* = 33)	PD‐NOH (*n* = 40)	*p* value
Middle cerebral artery velocity (cm/s)	68.93 ± 18.90	77.17 ± 20.15	.540
Resistance	0.58 ± 0.08	0.61 ± 0.06	.119
Third ventricle width (cm)	0.63 ± 0.19	0.60 ± 0.20	1.000
Level of the substantia nigra	3.00 ± 0.00	3.00 ± 0.00	–
Area of the substantia nigra (cm^2^)	0.34 ± 0.16	0.45 ± 0.18	.009*

Values are reported as the mean ± standard deviation (SD).

* *p *< .05 was considered statistically significant.

## 4 DISCUSSION

Our results showed that older age was associated with an increased prevalence of OH, which is consistent with previous studies (Palma et al., 2015; Szewczyk‐Krolikowski et al., 2014 ). There were no significant differences in gender, severity, or duration between the PD‐OH and PD‐NOH groups, although others have reported that disease duration is associated with OH in this population (Pilleri et al., 2013).

PD‐OH patients tend have lower scores in activities of daily living (ADL) than those with PD‐NOH (Mol et al., 2018). In PD‐OH patients, those with symptomatic OH tend to have lower ADL scores compared to those with asymptomatic OH group. This may be because symptomatic OH can cause dizziness and fainting (Kotagal et al., 2016). There was no difference in cognitive function between the symptomatic and asymptomatic PD‐OH groups.

We found that executive and visuospatial functions were statistically impaired in PD‐OH with OH compared with PD‐NOH. A previous study found that subjects with PD‐OH had impairments of specific functions, namely attention, visuospatial working memory, and verbal memory (Pilleri et al., 2013). This result partially overlaps with our conclusion. Another study found that executive function was reduced in the upright position (Sforza et al., 2018), which is consistent with our results.

The reasons underlying cognitive decline in PD‐OH patients is currently unclear. One hypothesis is that central and peripheral noradrenergic dysfunction may lead to cognitive deficits. Hypothalamus damage has been reported in PD patients (Langston & Forno, 1978). This structure is the integration center of the autonomic nervous system and is under the influence of the limbic system (Blessing, 1997). This may explain why executive functions were impaired in PD‐OH patients. Hemodynamics may play an important role in contributing to cognitive dysfunction. One study showed that hyperperfusion and hypotension may cause ischemic damage to subcortical structures (McDonald et al., 2016). Another group reported that cognitive impairment is associated with more white matter hyperintensities on magnetic resonance imaging (Kim et al., 2012). This is consistent with our findings; PD‐OH patients had higher SSP and lower OSP and ODP, and ischemic damage may affect executive function.

SN hyperechogenicity ≥0.20 cm^2^ is considered as a cut‐off point to detect PD (Chitsaz et al., 2013), but we found higher values in both groups in our study (PD‐OH: 0.34 cm^2^, PD‐NOH: 0.45 cm^2^). PD‐OH patients showed smaller SN hyperechogenic areas. It may indicate that different clinical subtypes of PD are associated with different SN patterns (Walter, Dressler, et al., 2007). Patients with postural instability and gait difficulty have larger SN hyperechogenicity areas on TCS (Sheng et al., 2017), so it is possible that PD patients with OH have smaller SN hyperechogenicity areas. TCS can also be used to diagnose parkinsonism. It is reported that the frequency of SN hyperechogenicity in nontremor dominant PD patients was significantly higher than in patients with multiple system atrophy (MSA) with predominant parkinsonism (Zhou et al., 2018). PD‐OH may be more likely to convert to MSA. The early presence of OH is a Movement Disorder Society clinical diagnostic criterion for PD (Postuma et al., 2015). Measuring the SN area may help us distinguish parkinsonism syndrome (PDS) from PD. However, it is not clear why the SN becomes hyperechogenic. Two studies reported that increased cellular iron and neuromelanin contents and microglia are associated with SN hyperechogenecity (Berg et al., 2010; Tribl et al., 2009 ). This may suggest different mechanisms underlying PD and PDS.

Our results should be considered in the context of its limitations. First, this was a cross‐sectional study, so when OH develops in patients with PD is not clear. Since we did not follow up for patient outcomes, the long‐term evolution of OH in PD remains unknown. Second, we did not adjust for dopaminergic therapy, which may influence cognitive function (McDonald et al., 2016). These therapies may also impact the presence and severity of OH, although one study reported that levodopa use was not significantly different between PD‐OH and PD‐NOH patients (McDonald et al., 2016).

## 5 CONCLUSION

In this study, age was an independent risk factor for OH in PD. The presence of OH had no influence on cognition. Only 31% of PD patients who meet OH diagnostic criteria are symptomatic (Palma et al., 2015). As a result, symptoms cannot be the only indicators to screen for OH. We often ignore asymptomatic OH in our clinical work, underscoring the need to test for OH in PD patients without symptoms. Treatment of neurogenic OH remains challenging because of the lack of clinical studies. Interventions must be applied in early stages of disease to improve cognition. TCS results were different between the PD‐OH and PD‐NOH groups, and the presence of OH may help distinguish different subtypes of PD or PDS. Further studies are necessary to confirm these findings.

## CONFLICT OF INTEREST

There is no conflict of interest to be disclosed.

### PEER REVIEW

The peer review history for this article is available at https://publons.com/publon/10.1002/brb3.2252.

## FUNDING INFORMATION

Social development projects in Jiangsu Province, Grant Number: BE2018658); Jiangsu Provincial Medical Key Discipline Project, Grant Number: ZDXKB2016022; Suzhou Science and Technology Development Program, Grant Number: SYS2020131; Discipline Construction Program of the Second Affiliated Hospital Soochow University, Grant Number: XKTJ‐XK202001.

## Data Availability

The data used to support the findings of this study are available from the corresponding author upon request.
